# Autopsy-based histopathological characterization of myocarditis after anti-SARS-CoV-2-vaccination

**DOI:** 10.1007/s00392-022-02129-5

**Published:** 2022-11-27

**Authors:** Constantin Schwab, Lisa Maria Domke, Laura Hartmann, Albrecht Stenzinger, Thomas Longerich, Peter Schirmacher

**Affiliations:** 1 Institute of Pathology, Heidelberg University Hospital, Universitätsklinikum Heidelberg, Pathologisches Institut, Im Neuenheimer Feld 224, 69120 Heidelberg, Germany; 2 German Center for Infection Research (DZIF), partner site Heidelberg, Institute of Pathology, Heidelberg University Hospital, Heidelberg, Germany

**Keywords:** Autopsy, SARS, Cardiac pathology, Myocarditis, Vaccination

## Abstract

Cases of myocarditis, diagnosed clinically by laboratory tests and imaging have been described in the context of mRNA-based anti-SARS-CoV-2 vaccination. Autopsy-based description of detailed histological features of vaccine-induced myocarditis is lacking. We describe the autopsy findings and common characteristics of myocarditis in untreated persons who received anti-SARS-CoV-2 vaccination. Standardized autopsies were performed on 25 persons who had died unexpectedly and within 20 days after anti-SARS-CoV-2 vaccination. In four patients who received a mRNA vaccination, we identified acute (epi-)myocarditis without detection of another significant disease or health constellation that may have caused an unexpected death. Histology showed patchy interstitial myocardial T-lymphocytic infiltration, predominantly of the CD4 positive subset, associated with mild myocyte damage. Overall, autopsy findings indicated death due to acute arrhythmogenic cardiac failure. Thus, myocarditis can be a potentially lethal complication following mRNA-based anti-SARS-CoV-2 vaccination. Our findings may aid in adequately diagnosing unclear cases after vaccination and in establishing a timely diagnosis in vivo, thus, providing the framework for adequate monitoring and early treatment of severe clinical cases.

**Graphical abstract:**

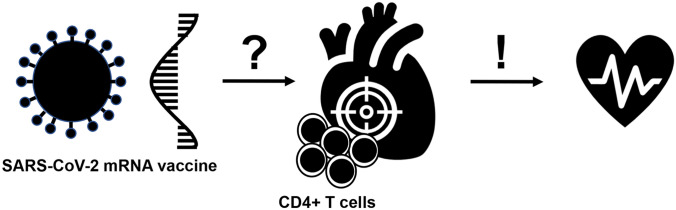

**Supplementary Information:**

The online version contains supplementary material available at 10.1007/s00392-022-02129-5.

## Introduction

Between December 2020 and March 2021, the European Medicines Agency approved several vaccines on the basis of randomized, blinded, controlled trials: two messenger RNA-based vaccines—Comirnaty, BNT162b2 (Pfizer–BioNTech) and Spikevax, mRNA-1273 (Moderna)—both encoding the spike protein of SARS-CoV-2 encapsulated in lipid nanoparticles as the antigen and two vaccines based on recombinant adenoviruses (Vaxzevira, ChAdOx1 nCov-19 (AstraZeneca), a recombinant chimpanzee adenoviral vector encoding the spike glycoprotein of SARS-CoV-2 and Ad26.COV2.S (Johnson & Johnson/Janssen), a recombinant adenovirus type 26 vector encoding SARS-CoV-2 spike glycoprotein. Recently, the first adapted bivalent COVID-19 booster vaccines targeting Omicron subvariants (BA.1 and BA.4-5, respectively) were authorized across the European Union (EMEA/H/C/005735: Comirnaty Original/Omicron BA.1, Comirnaty Original/Omicron BA.4-5; EMEA/H/C/005791: Spikevax bivalent Original/Omicron BA.1).

As vaccines may cause adverse events (AEFI), it is crucial to record them systematically and assess them for causality both at the population and at the individual level, as proposed by the World Health Organization (WHO) [1]. Detailed analyses should aim to establish or rule out a causal link between vaccination and the event in question. Autopsy is an important measure to identify severe adverse effects and to provide important mechanistic data in this setting. It may allow to identify the population at risk and may help to develop algorithms for prevention or monitoring, facilitating early diagnosis and successful treatment.

Cases of (epi-)myocarditis have previously been documented after immunization against smallpox or influenza in the vaccine adverse events reporting system [2, 3]. Recently, unusual cases of (epi-)myocarditis after vaccination with mRNA-based anti-SARS-CoV-2-vaccines have been documented [4]. These were clinically observed and diagnosed by laboratory and cardiac magnetic resonance imaging, predominantly in males under 30 years of age [5–8]. Available short-term follow-up data suggest resolution of symptoms [5–7]. However, few individuals required intensive care support or even died from acute heart failure. Information about potential long-term health outcomes is not yet available. Verma et al. reported two cases of myocarditis after mRNA vaccination, one of them fatal, revealed by endomyocardial biopsy and autopsy respectively [9]. Histology showed an inflammatory infiltrate predominantly composed of T-cells and macrophages, admixed with eosinophils, B-cells and plasma cells. By reporting similar observations based on different diagnostic techniques (e.g. cardiac magnetic resonance imaging, endomyocardial biopsy), the causality of an potential AEFI can be assessed at the population level [1]. However, in most of these studies comprehensive testing for infectious agents, crucial for the assessment of an AEFI at the individual level, was not reported. As a consequence, a systematic description with histopathological phenotyping as well as molecular analysis of (epi-)myocarditis after anti-SARS-CoV-2-vaccination is still lacking.

Here, we describe the cardiac autopsy findings in five persons who have died unexpectedly within seven days following anti-SARS-CoV-2-vaccination, with vaccine-induced myocardial inflammation representing the likely or possible cause of death. Our findings establish the histological phenotype of lethal vaccination-associated myocarditis.

## Materials and methods

Data on autopsies of persons, who received anti-SARS-CoV-2 vaccination (up to 20 days before their death), were obtained from the COVID autopsy and biomaterial registry Baden-Württemberg. This federal state registry contains autopsy, clinical and pathological data as well as tissue samples from patients who have died in the context of a SARS-CoV-2 infection or persons who have died briefly after an anti-SARS-CoV-2 vaccination (*n* = 54). All autopsies were performed at one of the five University hospital sites (Heidelberg, Tübingen, Freiburg, Ulm, Mannheim) of Baden-Württemberg. The network cooperates with prosecution and the forensic pathology as well as with other national networks such as the German Center for Infection Research (DZIF), the German Center for Lung Research (DZL), and the national academic research network NUM (Network of University Medicine; DEFEAT PANDEMIcs); results are constantly reported to the Paul Ehrlich Institute (PEI), the German Federal Institute for Vaccines and Biomedicines. In this study, all and only autopsies performed at Heidelberg University Hospital (*n* = 35) were included to ensure that all medical documents and findings were available and that the autopsies were performed according to the standardized procedure described previously [10]. The hearts were examined macroscopically by measuring the weight and the thickness of the left, right and interventricular walls. Coronary arteries were dissected from their aortic branching to the periphery to allow for evaluation of arteriosclerosis and exclusion of thrombi. Afterwards, the inflow and outflow tracts were examined, the ventricles were cross-sectioned in short axis (transversal plane) at 1 cm intervals from the valves to the apex and the cut surfaces were examined for focal lesions (or geographic demarcations), a focussed dissection and detailed histological evaluation of the cardiac conduction system was not performed.

For histological evaluation, all tissue samples were fixed in 4% neutral-buffered formalin. At least two full-thickness blocks from the left and right ventricular wall, the interventricular septum, and the papillary muscles were taken for histological evaluation of cardiac pathologies. From formalin-fixed, paraffin-embedded (FFPE) tissue-blocks at least two sections with a thickness of 4 µm were stained with hematoxylin & eosin, periodic acid Schiff (PAS) reaction, and acid fuchsin orange G (AFOG), respectively. Immunohistochemical stainings were performed according to standard protocols. In brief, immunohistochemistry was performed on an automated immunostainer (Ventana BenchMark Ultra, Ventana Medical Systems, Tucson, USA). Sections were cut, deparaffinized, rehydrated and pre-treated with an antigen retrieval buffer (Tris/Borat/EDTA, pH 8.4). After blocking of endogenous peroxidase, the slides were incubated with monoclonal antibodies directed against CD3 (clone 2GV6, Roche, Rotkreuz, Switzerland), CD4 (clone SP35, Roche), CD8 (clone SP57, Roche), CD20 (clone L26, Roche), GATA 3 (clone L50-823, Roche, Rotkreuz, Switzerland) and D2-40 (clone Roche, Rotkreuz, Switzerland) at the provided dilutions of the ready-to-use-kits, CD68 (clone PgM1, Agilent/Dako, Santa Clara, United States) at a dilution of 1:100, FOXP3 (clone D2W8ETM, Cell iSignaling Technology, Danvers, MA, United States) at a dilution of 1:25, and Tbet (clone 4B10, Santa Cruz Biotechnology Inc., Santa Cruz, CA, United States) at a dilution of 1:50 followed by incubation with OptiView Universal Linker and OptiView HRP Multimer. Visualization was achieved using DAB as chromogen. Before mounting, slides were counterstained with haematoxylin. Histological and immunohistological findings were analysed in synopsis with available data from the patients’ medical records. Three age- and sex-matched cohorts from our autopsy files (covering the years 2005/2006, 2010/2011 and 2015/2016) were retrieved and the myocardial samples were evaluated for the presence and phenotype of inflammatory infiltrates.

Based on the Dallas criteria and the specifications according to Caforio et al. myocarditis was defined by an inflammatory infiltrate with ≥ 14 leucocytes/mm^2^ including up to 4 monocytes/mm^2^ with the presence of CD3 positive T-lymphocytes ≥ 7 cells/mm^2^ and signs of myocyte degeneration and necrosis of non-ischaemic origin [11, 12]. Myocardial and epicardial infiltration was assessed semiquantitatively by visual scoring using a four-tiered system (0–3): score 0 (no foci of inflammation), score 1 (focal, isolated foci with up to 20 leucocytes/mm^2^), score 2 (focal, > 20 leucocytes/mm^2^), and score 3 (diffuse infiltration). Regarding potential causative infectious agents, FFPE samples of all cases and fresh frozen myocardial samples of cases 1, 3, 4 and 5 were tested for viral and bacterial genomes by a diagnostic panel with (reverse transcription) PCR (Enteroviruses, parvovirus B19, human herpesvirus 6, Epstein–Barr virus, human cytomegalovirus, varicella zoster virus, herpes simplex virus type 1 and 2, human herpesvirus 7, adenoviruses, borrelia spp., Toxoplasma gondii). For each sample, 350 ng of total nucleic acid was extracted and subjected to nested (RT)-PCR using in-house techniques as described by Mahfoud et al. [13]. Glyceraldehyde-3-phosphate dehydrogenase (GAPDH) RT-PCR served as an internal control for both nucleic acid extraction and PCR amplification.

The likelihood of vaccine-induced (epi-)myocarditis was categorized according to the criteria detailed in Table 1.

**Table 1:** Likelihood assessment of vaccine-induced (epi-)myocarditis

category	Criteria
No vaccine-induced myocarditis	- Absence of myocarditis OR - Myocarditis without temporal association to vaccination event OR - Myocarditis, definitely explained by other, especially infectious diseases defined by histological/microbiological/virological testing of cardiac tissue
Possible vaccine-induced myocarditis	- Presence of myocarditis with temporal association to vaccination event, but presence of mitigating factors (e.g. detection of facultatively pathogenic infectious agents) OR - Presence of pericarditis with temporal association to vaccination event, but myocardial findings insufficient to establish definite myocarditis (e.g. Dallas criteria)
Likely vaccine-induced myocarditis	- Presence of myocarditis with temporal association to vaccination event AND - Integration of histological phenotype, clinical presentation, and laboratory findings indicate no alternative differential diagnosis

## Results

Among the 35 cases of the University of Heidelberg, autopsies revealed other causes of death (due to pre-existing illnesses) in 10 patients (Supplementary Table 1). Hence, these were excluded from further analysis. Cardiac autopsy findings consistent with (epi-)myocarditis were found in five cases of the remaining 25 bodies found unexpectedly dead at home within 20 days following SARS-CoV-2 vaccination. Main characteristics of the five cases are presented in Table 2, while further autopsy findings are shown in Supplementary Table 2. Three of the deceased persons were women, two men. Median age at death was 58 years (range 46–75 years). Four persons died after the first vaccine jab, the remaining case after the second dose. All persons died within the first week following vaccination (mean 2.5 days, median 2 days). Clinical findings, blood tests, ECGs or imaging data were not available as deceased persons did not seek medical attention prior to death. Person 1 was found dead 12 h after the vaccination. A witness described a rattling breath shortly before discovering circulatory failure. Person 2 complained about nausea and was found dead soon thereafter. Resuscitation was started immediately but without success, respectively. The other persons were found dead at home without available information about terminal symptoms. According to the available information provided at the time of autopsies, none of the deceased persons had SARS-CoV-2 infection prior to vaccination and nasopharyngeal swabs were negative in all cases.

**Table 2:** Case characteristics

Case	Gender	Age	BMI	Vaccine type	Dose	Time from vaccination to death (days)	Time from death to autopsy (days)	Comorbidity	Grading – myocarditis (0 – 3)	Grading – epicarditis (0 – 3)	PCR analysis	Assessment of causal relationsship
1	male	46	31.8	Cormirnaty (BioNTech)	First	0	7	AH	2	0	Negative	Likely
2	female	50	20	Spikevax (Moderna)	First	1	3	-	1	2	Negativ	Likely
3	female	62	22.5	Cormirnaty (BioNTech)	First	7	3	COPD	1	1	Negative	Possible
4	male	55	30.1	Cormirnaty (BioNTech)	Second	4	3	-	2	3	Negative	Likely
5	female	75	27,9	Cormirnaty (BioNTech)	First	1	9	AH, DM, Hashimoto´s thyroiditis	2	2	HHV6	Possible

Abbreviations: *AH* arterial hypertension, *COPD* chronic obstructive pulmonary disease, *DM* diabetes mellitus

**Fig. 1:**
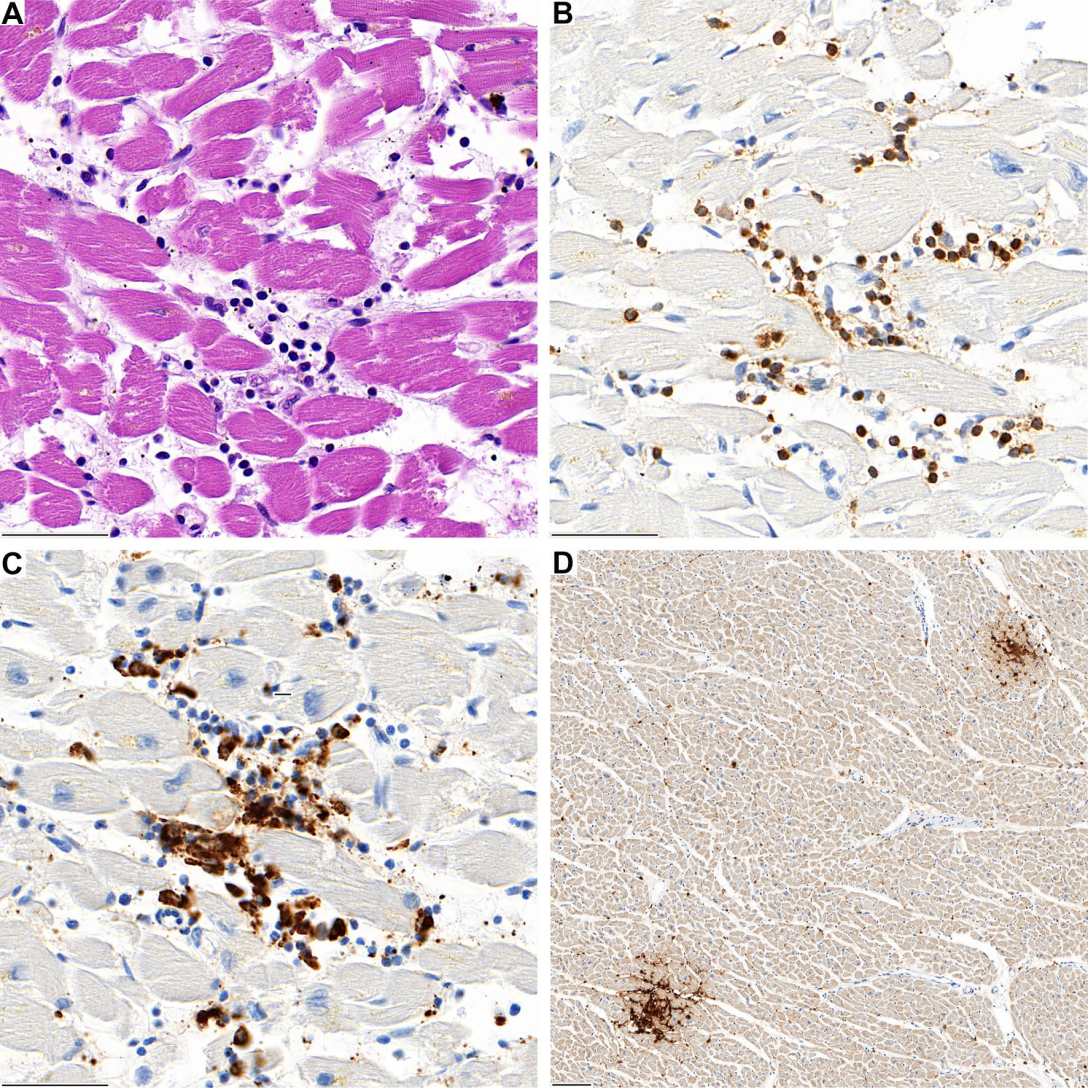
**A** Lymphocytic aggregates in the interventricular septum of case 1 with associated myocardiocyte destruction. **B** The infiltrate is predominantly composed of CD3-positive T-lymphocytes and **C** CD68-positive macrophages. **D** In lower magnification two foci of CD4-positive lymphocytes are evident (**D**)

**Fig. 2:**
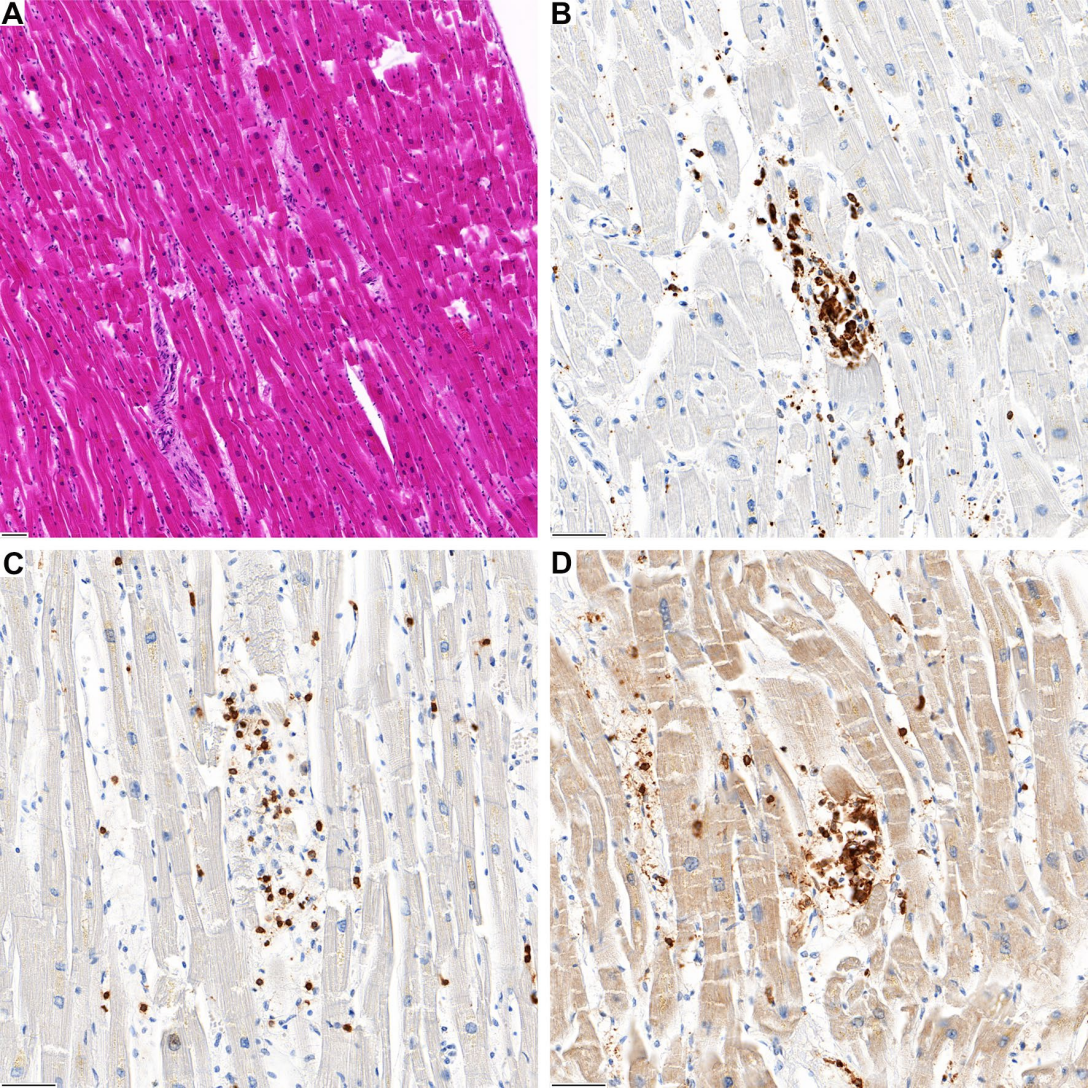
**A** Inflammatory focus in the left ventricular wall of case 2. **B** The infiltrate is predominantly composed of CD68-positive macrophages and **C** CD3-positive T-lymphocytes with (**D**) co-expression of CD4

Histological examination showed inflammatory infiltration of the myocardium. The infiltrate was focal and interstitial in all cases. It was predominantly detected in sections taken from the right ventricular wall and interventricular septum. The histological and immunohistochemical characterization revealed that the inflammatory infiltrate was predominantly composed of lymphocytes. The number of CD3-positive T-cells by far outnumbered the few CD20-positive B-cells detected. In addition, most T-cells belonged to the CD4-positive subset, while only scattered CD8-positive T-cells were seen (Fig. 1, 2, Supplementary Fig. 1/2). The T cells were negative for Tbet as a marker for Th1 cells, GATA3 as a marker for Th2 cells, D2-40, as a marker for Th17 cells (Supplementary Fig. 2). In addition, FOXP3 positive regulatory T cells and CD21 positive follicular dendritic cells were not detected within the cardiac infiltrates, while control cases, including cases of sarcoidosis were positive (Supplementary Fig. 2/3). Immunohistochemistry for CD68 showed few interspersed histiocytes (Fig. 2, Supplementary Fig. 1). Microfocal myocyte injury was demonstrable in three cases (patient 1, 2 and 3). No granulomas were found. All cases lacked significant coronary heart disease, acute or chronic manifestations of ischaemic heart disease, manifestations of cardiomyopathy or other signs of a pre-existing, clinically relevant heart disease.

**Fig. 3:**
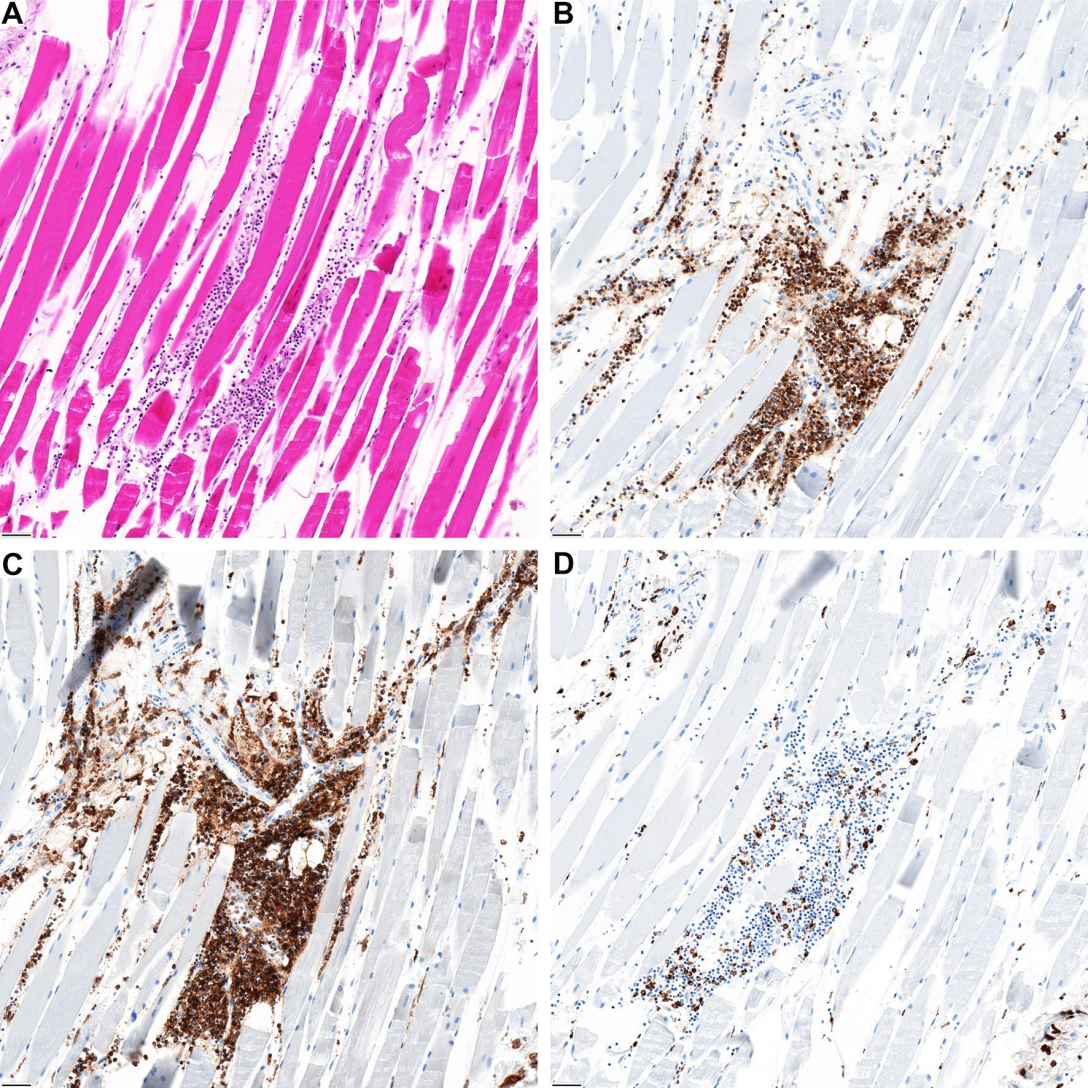
**A** The jab site in the deltoid muscle reveals focal inflammation. The composition is similar to the phenotype of the myocardial infiltrates showing predominantly, **B** CD3 and **C** CD4-coexpressing lymphocytes and **D** interspersed CD68-positive macrophages

In most cases, an inflammatory infiltration of the epicardium and the subepicardial fat tissue was concomitantly found (cases 2, 3, 4 and 5; Supplementary Fig. 4) and revealed an identical immunophenotype. (T-cell dominant; CD4 >  > CD8). In case 2, a prominent CD4-positive lymphocytic infiltration was also recorded at the jab site of the deltoidal muscle (Fig. 3). Analysis for potential infectious agents causing a myocarditis revealed low viral copy numbers of human herpes virus 6 (HHV6) in one case (case 5). The results of the other four cases were negative for all infectious agents tested, but demonstrated regular amplification of the GAPDH control suggesting adequate nucleic acid quality for analysis.

In three cases, the overall autopsy findings, in particular presence of (epi-)myocarditis in combination with the absence of other plausible causes of death (especially pulmonary embolism, myocardial infarction, severe brain infarction or bleeding, other cardiac disease), together with the close temporal association with the vaccination event lead to the conclusion that vaccination was the likely cause of (epi-)myocarditis and that this cardiac affection was the cause of sudden death. For case 5, myocarditis was considered to be the cause of death as well, but the detection of HHV6, even in low viral copy numbers provided an alternative explanation for the presence of myocarditis. With regard to the question of a fatal AEFI, case 5 was therefore classified as “possible”. For case 3 no other cause for the inflammatory infiltration was found, but the infiltrate was discrete and mainly observed in the pericardial fat. Thus, case 3 was categorized as possible AEFI as well. We did not find an obvious association between the infiltrates and endothelial cells (CD31, D2-40), mesothelial cells (calretinin), or neural cells (S100). During the last 20 years of autopsy service at Heidelberg University Hospital we did not observe comparable myocardial inflammatory infiltration. This was validated by histological re-evaluation of age- and sex-matched cohorts from three independent periods, which did not reveal a single case showing a comparable cardiac pathology.

## Discussion

Several cases of myocarditis following anti-SARS-CoV-2 vaccination have been published [4–6, 9, 14]. Symptoms typically occur within the first three days following the second dose of mRNA COVID-19 vaccines (Comirnaty and Spikevax, respectively) and young male patients presenting with chest pain are predominantly affected. Clinical findings like elevated troponin serum levels, abnormal ST-elevations in ECG and altered ventricle movement in echocardiogram or late enhancement in cardiac MRI suggested the development of a myocarditis. Most of the reported cases showed clinically mild courses with resolution of symptoms without treatment. However, in rare instances individuals required intensive care support or even died from acute heart failure as described in an early report by Verma et al. [9]. These studies, with their different diagnostic modalities applied, already pointed to a link between vaccination and myocarditis, though many of these studies lack extensive testing for infectious agents. In particular studies of autopsy cohorts as well as information about potential long-term outcomes are not available yet [15–17].

Through our autopsy-based approach, we identified five cases of lymphocytic (epi-)myocarditis in persons, who were unexpectedly found dead at home within the first week following mRNA-mediated anti-SARS-CoV-2 immunization. According to the Dallas criteria four samples were classified as definitive myocarditis. In the remaining case, comparable inflammatory infiltration of the epicardium, subepicardial fat and myocardium was found, but myocardial infiltration did not exceed the threshold of the Dallas criteria. All cases showed a consistent phenotype: (A) focal interstitial lymphocytic myocardial infiltration, in three cases accompanied by demonstrable microfocal myocyte destruction. (B) T-cell dominant infiltrate with CD4 positive T-cells outnumbering CD8 positive T-cells by far; (C) frequently associated with T-cell infiltration of epicardium and subepicardial fat tissue revealing a similar immune phenotype (CD4 >  > CD8).

As well-known from myocardial infarction, it has to be considered that microscopically visible manifestation of myocardial damage under such acute conditions may lag behind function; this may relate to aspects of infiltrate composition, such as the relatively low macrophage content, or the histologically focal myocyte damage. Thus, functional effects may be much stronger than expected considering the histological picture. This is reflected by the fact that myocarditis is a major cause of sudden and unexpected death in infants, adolescents, and young adults with frequencies ranging from 1 to 14% among the young [18–21]. As outlined in the materials and methods section, evaluation of the likelihood of an AEFI reflects the temporal association and the autopsy findings (with exclusion of other reasons of sudden death), and negative molecular testing for potential infectious causes. Thus, case 5 with HHV6-DNA detected at low copy numbers was classified as possible. In general, a causal link between myocarditis and anti-SARS-CoV-2 vaccination is supported by several considerations: (A) a close temporal relation to vaccination; all cases were found dead within one week after vaccination, (B) absence of any other significant pre-existing heart disease, especially ischaemic heart disease or cardiomyopathy, (C) negative testing for potential myocarditis-causing infectious agents, (D) presence of a peculiar CD4 predominant T-cell infiltrate, suggesting an immune mediated mechanism. The latter criterion is supported by demonstration of a phenotypically identical T-cell infiltrate at the deltoidal injection site in one of the cases. It has to be emphasized, that a comparable (epi-)myocardial infiltration was neither found in any of the other 20 autopsies performed on bodies found dead within 20 days following an anti-SARS-CoV-2 vaccination nor in the age- and sex-matched cohorts from three independent periods from our autopsy-files.

Based on the autopsy findings and all available data, no other cause of death except (epi-)myocarditis was identified in any of the cases presented here. Hence, myocarditis has to be considered the likely cause of death. From a functional point of view, myocardial damage in our cases is not sufficient to postulate contractile failure as terminal cause of death; thus, arrhythmic failure, either by cardiac arrest or by ventricular fibrillation, has to be assumed as the mechanism leading to the patients’ death. Myocarditis-related acute cardiac arrest due to either asystoly or ventricular fibrillation is a well-established pathomechanism in other causes of acute myocarditis as well [22, 23].

Regarding the potential underlying pathogenesis of (epi-)myocarditis, our findings allow some considerations. Besides pneumonia, myocarditis is another manifestation reported during SARS-CoV-2-infection [24]. It is under debate whether myocarditis in COVID-19 is primarily caused by the viral infection or whether it occurs secondary as a consequence of the host´s immune response, in particular by T-lymphocyte-mediated cytotoxicity or as a consequence of the cytokine storm observed during COVID-19 [25]. Thus, it seems possible that a molecular mimicry between the spike protein of SARS-CoV-2 and self-antigens may trigger an anti-myocytic immune response in predisposed individuals. Multiple studies of mRNA-vaccines showed robust Receptor-Binding-Domain specific antibodies, T cell and cytokine responses [26]. T cells, especially CD4 + T cells, are the main drivers of heart-specific autoimmunity in myocarditis [27]. A vaccine-induced activation of the immune system in persons with otherwise peripheral tolerance due to regulatory T cells might promote CD4 + effector T cell expansion and myocarditis. Considering that (epi-)myocarditis has not been described following vector-based anti-SARS-CoV-2 immunization yet, it could also be possible that the immune response may be directed against the mRNA or other constituents of the vaccine formula. However, the vaccine against smallpox, based on a vaccinia virus, is reported to cause (epi-)myocarditis in rare cases [2, 3]. Of note, it has been recently reported that intravenous injection of COVID-19 mRNA vaccine is able to induce an acute (epi-) myocarditis in a preclinical model [28]. Interestingly, we recorded inflammatory foci predominantly in the right heart, which may suggest a gradual blood-stream derived dilution effect and based on this finding it is at least tempting to speculate that inadvertent intravascular vaccine injection may be contributive.

Our study is limited by the relatively small cohort size and inherits the bias of an endpoint analysis. The nature of our autopsy study necessitates that the data are descriptive in quality and does not allow any epidemiological conclusions in terms of incidence or risk estimation. The reported incidence of (epi-)myocarditis after vaccination is low and the risks of hospitalization and death associated with COVID-19 are stated to be greater than the recorded risk associated with COVID-19 vaccination [29]. Importantly, infectious agents may also cause lymphocytic myocarditis with a similar immunophenotype, thus meticulous molecular analyses is required in all cases of potentially vaccination-associated myocarditis.

Regarding a potential auto-immunological mechanism explaining the myocardial damage, histological examination of lymphatic nodes might be of interest, as Röltgen et al. described altered germinal center architecture following COVID-19 vaccination [30]. This aspect could not be addressed in our analysis, as systematic lymph node sampling was not part of our standardized autopsy protocol.

Finally, we cannot provide a definitive functional proof or a direct causal link between vaccination and myocarditis. Further studies and extended registry are needed to identify persons at risk for this potentially fatal AEFI and may be aided by detailed clinical, serological, and molecular analyses which were beyond the scope of this study. Considering that this fatal adverse event may affect healthy individuals, such registry and surveillance programs may improve early diagnosis, close monitoring, and treatment.

## Supplementary Information

Below is the link to the electronic supplementary material.

Supplementary file1 (DOCX 18 KB)

Supplementary file2 (DOCX 15 KB)

**Supplementary Figure 1**: (A) Inflammatory infiltrate in the left ventricular wall of case 5. (B) Most of the CD3-positive T-lymphocytes reveal (C) coexpression of CD4. (D) Again CD68-positive macrophages belong to the inflammatory infiltrate (TIFF 17020 KB)

**Supplementary Figure 2**: Inflammatory infiltrate in the myocardium of case 4. (A) The infiltrate is predominantly composed of CD3-positive T-lymphocytes with (B) CD4-positive cells by far outnumbering (C) CD8-positive lymphocytes. (D) Few CD68-positive macrophages are also seen. The T cells do neither express (E) Tbet, a marker for Th1 cells, (F) nor GATA3, a marker for Th2 cells, nor (G) D2-40, a marker for Th17 cells, nor (H) FOXP3-positive regulatory T cells (TIFF 10508 KB)

**Supplementary Figure 3**: Control case showing lymph node involvement by sarcoidosis. (A) Numerous CD3-positive lymphocytes surrounding epithelioid granulomas are ssen. (B) CD4-positive cells outnumber (C) CD8-positive lymphocytes, while (D) CD68 highlights the epithelioid cells within the granuloma. (E) Only few T cells express Tbet, while (F) GATA3 is weakly expressed by many T-cells. (G) Neo D2-40-positive cells are detected, while (H) FOXP3 detects the the presence of regulatory T cells in the lymphcytic infiltrate and the granuloma (TIFF 11162 KB)

**Supplementary Figure 4**: (A) Inflammatory infiltration of the epicardium of case 4, again showing (B) numerous CD68-positive macrophages and a T-cell infiltrate with (C) CD4-positive lymphocytes outnumbering (D) CD8-positive lymphocytes (TIFF 15031 KB)

## Data Availability

Data are available upon reasonable request.
