# Supplementary material for: Autopsy-based histopathological characterization of myocarditis after anti-SARS-CoV-2-vaccination
Source: Clin Res Cardiol. 2022 Nov 27;112(3):431–40. doi: 10.1007/s00392-022-02129-5 (PMC9702955; doi:10.1007/s00392-022-02129-5)
Supplement: Supplementary file 1 — Supplementary file1 (DOCX 18 KB) [file 392_2022_2129_MOESM1_ESM.docx]

**Supplementary Table 1: autopsy findings for the cases 6 - 35**

| **case** | **age** | **sex** | **pre-existing diseases** | **cause of death** |
| --- | --- | --- | --- | --- |
| 6 | 73 | male | ischemic cardiomyopathy | arrhythmogenic cardiac failure |
| 7 | 56 | male | ischemic cardiomyopathy | arrhythmogenic cardiac failure |
| 8 | 64 | male | METS, CAD | COVID-pneumonia |
| 9 | 78 | female | AH, COPD, | cardiac failure |
| 10 | 70 | male | AH, CAD, organising pneumonia | cardiac failure, pneumonia, pulmonary embolism |
| 11 | 61 | female | CAD | stroke |
| 12 | 31 | male | AH, astma, cardiac hypertrophy | cardiac failure |
| 13 | 75 | male | AH, CAD, COPD, prostate cancer | myocardial infarction |
| 14 | 59 | male | AH, CAD, ischemic cardiomyopathy | cardiac failure |
| 15 | 77 | male | AH, CAD, DM2, COPD, metastatic colon cancer | respiratory failure |
| 16 | 63 | male | DM2, AH, CAD, parkinsons disease | bronchopneumonia |
| 17 | 84 | male | AH, DM2 | paralytic ileus |
| 18 | 38 | male | no relevant preexisting disease, vaccination with ChAdOx1 nCov-19 | vaccine-induced thrombotic thrombocytopenia |
| 19 | 49 | female | not applicable | myocardial infarction |
| 20 | 68 | male | DM2, AH, hypothyreosis | cerebral mass hemorrhage |
| 21 | 75 | female | artrial fibrillation, CKD | ruptured aortic aneurysm |
| 22 | 23 | female | no relevant preexisting disease | pulmonary embolism |
| 23 | 63 | female | not applicable | right heart failure, deep vein thrombosis |
| 24 | 70 | male | not applicable | myocardial infarction |
| 25 | 30 | female | drug abuse | intoxication |
| 26 | 39 | male | not applicable | cardiac tamponade |
| 27 | 57 | female | AH, CAD, ischemic cardiomyopathy | arrhythmogenic cardiac failure |
| 28 | 21 | male | drug abuse | intoxication |
| 29 | 69 | male | CAD | myocardial infarction |
| 30 | 21 | male | astma, cardiac hypertrophy | cardiac failure |
| 31 | 30 | male | drug abuse | bronchopneumonia |
| 32 | 55 | male | no relevant preexisting disease | chronic cardiomyopathy |
| 33 | 26 | male | drug abuse | intoxication |
| 34 | 31 | female | not applicable | ruptured aneurysm of carotid artery |
| 35 | 63 | male | DM2, gout | myocardial infarction |

Abbreviations: AH, arterial hypertension; CAD, coronary artery disease; COPD, chronic obstructive pulmonary disease; DM2, diabetes mellitus type 2.
