# Supplementary material for: Autopsy-based histopathological characterization of myocarditis after anti-SARS-CoV-2-vaccination
Source: Clin Res Cardiol. 2022 Nov 27;112(3):431–40. doi: 10.1007/s00392-022-02129-5 (PMC9702955; doi:10.1007/s00392-022-02129-5)
Supplement: Supplementary file 2 — Supplementary file2 (DOCX 15 KB) [file 392_2022_2129_MOESM2_ESM.docx]

**Supplementary Table 2: Reported clinical presentation and additional findings during autopsy**

|  | | **Autopsy** **findings** | | | | | | | |
| --- | --- | --- | --- | --- | --- | --- | --- | --- | --- |
| **case** | **Clinical presentation** | **Vascular system** | **Lungs** | **Hepato-pancreato-biliary system** | **Gastrointestinal system** | **Urogenital system** | **Endocrine system** | **Hemato-lymphoid system** | **Cerebral system** |
| 1 | circulatory failure following rattling breath | mild atherosclerosis | congestion, alveolar edema | no relevant pathology | no relevant pathology | benign nephrosclerosis | adrenal cortical adenoma | no relevant pathology | no relevant pathology |
| 2 | nausea | mild atherosclerosis | congestion, alveolar edema | mild chronic pancreatitis | no relevant pathology | benign nephrosclerosis | no relevant pathology | no relevant pathology | no relevant pathology |
| 3 | not reported | severe atherosclerosis | COPD | focal nodular hyperplasia | no relevant pathology | benign nephrosclerosis | no relevant pathology | no relevant pathology | no relevant pathology |
| 4 | not reported | moderate atherosclerosis | sarcoidosis | sarcoidosis | no relevant pathology | no relevant pathology | no relevant pathology | no relevant pathology | no relevant pathology |
| 5 | not reported | moderate atherosclerosis | lymphocytic capillaritis | no relevant pathology | no relevant pathology | no relevant pathology | Hashimoto´s thyroiditis | no relevant pathology | status following infarction (7 years ago) |

Abbreviation: COPD, chronic obstructive pulmonary disease
