# Supplementary figures and images for: Autopsy-based histopathological characterization of myocarditis after anti-SARS-CoV-2-vaccination
Source: Clin Res Cardiol. 2022 Nov 27;112(3):431–40. doi: 10.1007/s00392-022-02129-5 (PMC9702955; doi:10.1007/s00392-022-02129-5)

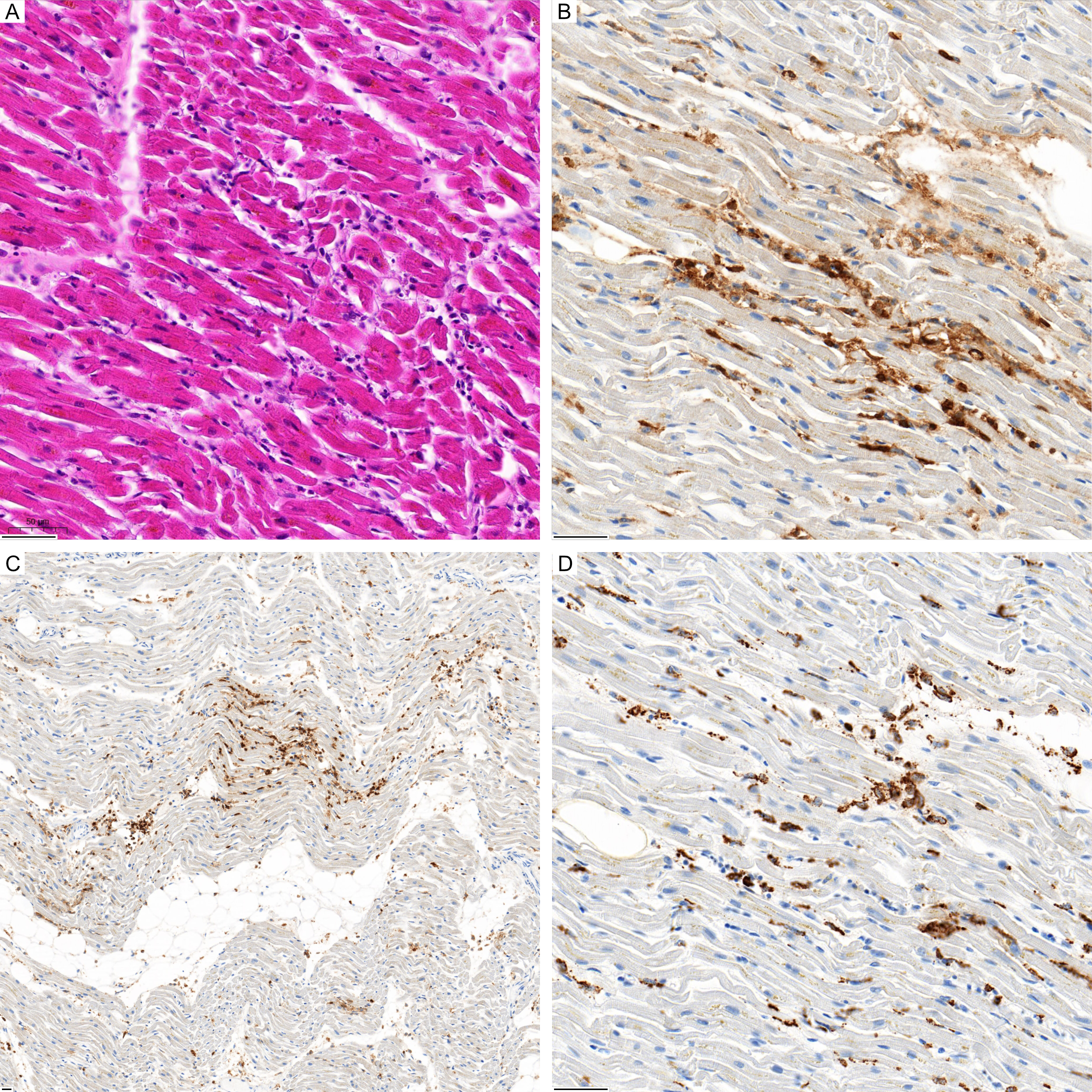

Supplement: Supplementary file 3 — Supplementary Figure 1: (A) Inflammatory infiltrate in the left ventricular wall of case 5. (B) Most of the CD3-positive T-lymphocytes reveal (C) coexpression of CD4. (D) Again CD68-positive macrophages belong to the inflammatory infiltrate (TIFF 17020 KB) [file 392_2022_2129_MOESM3_ESM.tiff]

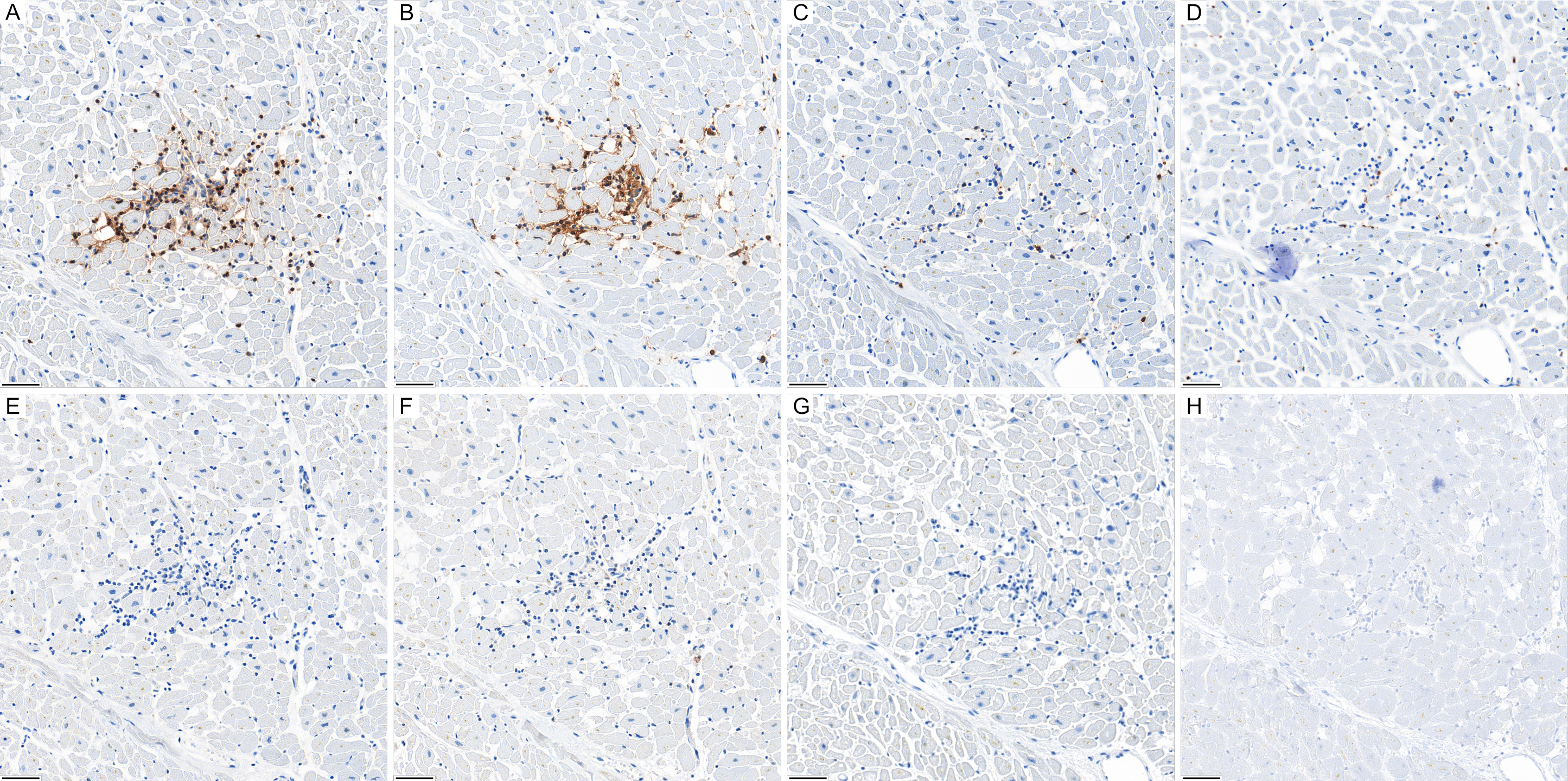

Supplement: Supplementary file 4 — Supplementary Figure 2: Inflammatory infiltrate in the myocardium of case 4. (A) The infiltrate is predominantly composed of CD3-positive T-lymphocytes with (B) CD4-positive cells by far outnumbering (C) CD8-positive lymphocytes. (D) Few CD68-positive macrophages are also seen. The T cells do neither express (E) Tbet, a marker for Th1 cells, (F) nor GATA3, a marker for Th2 cells, nor (G) D2-40, a marker for Th17 cells, nor (H) FOXP3-positive regulatory T cells (TIFF 10508 KB) [file 392_2022_2129_MOESM4_ESM.tiff]

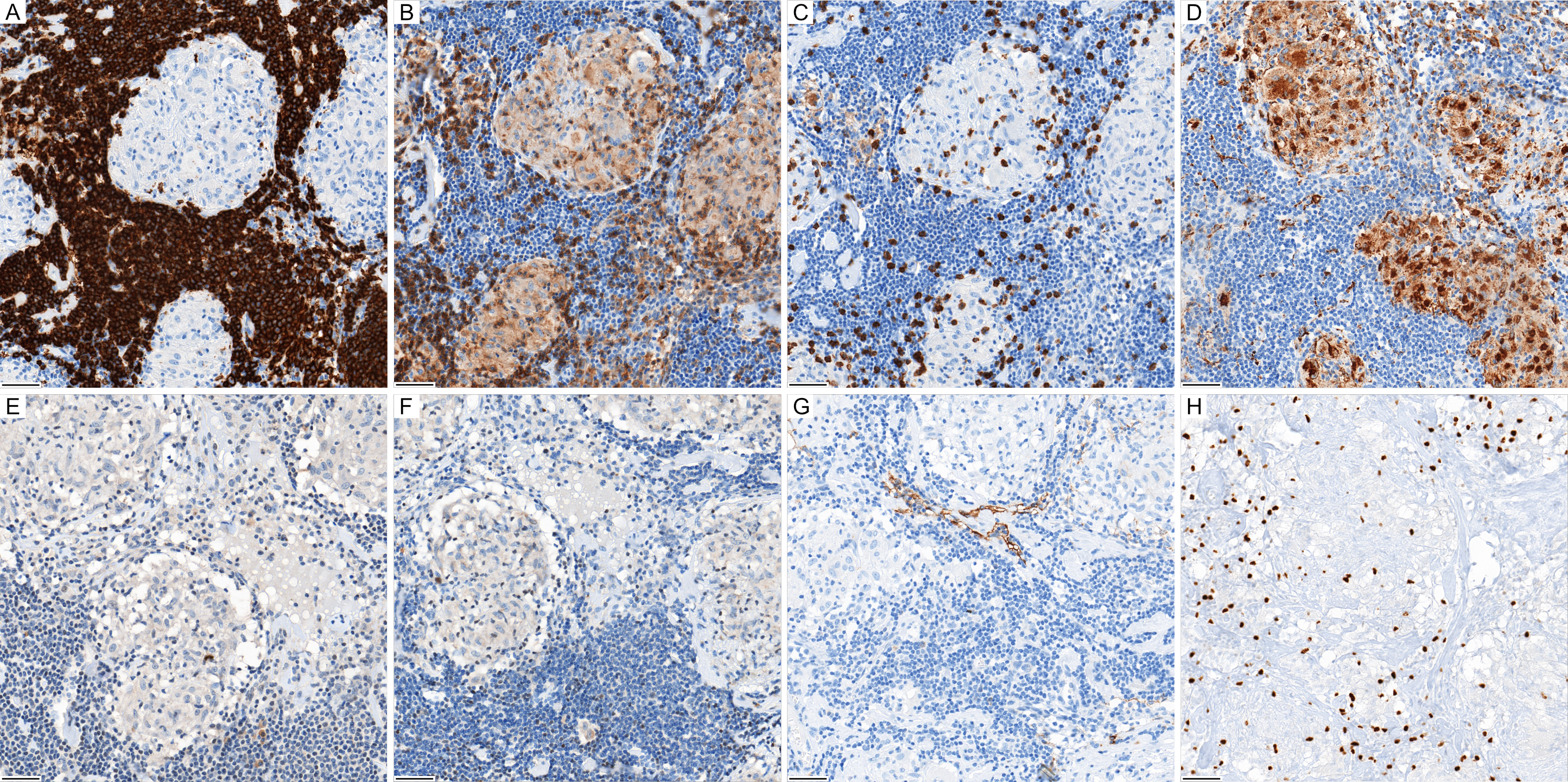

Supplement: Supplementary file 5 — Supplementary Figure 3: Control case showing lymph node involvement by sarcoidosis. (A) Numerous CD3-positive lymphocytes surrounding epithelioid granulomas are ssen. (B) CD4-positive cells outnumber (C) CD8-positive lymphocytes, while (D) CD68 highlights the epithelioid cells within the granuloma. (E) Only few T cells express Tbet, while (F) GATA3 is weakly expressed by many T-cells. (G) Neo D2-40-positive cells are detected, while (H) FOXP3 detects the the presence of regulatory T cells in the lymphcytic infiltrate and the granuloma (TIFF 11162 KB) [file 392_2022_2129_MOESM5_ESM.tiff]

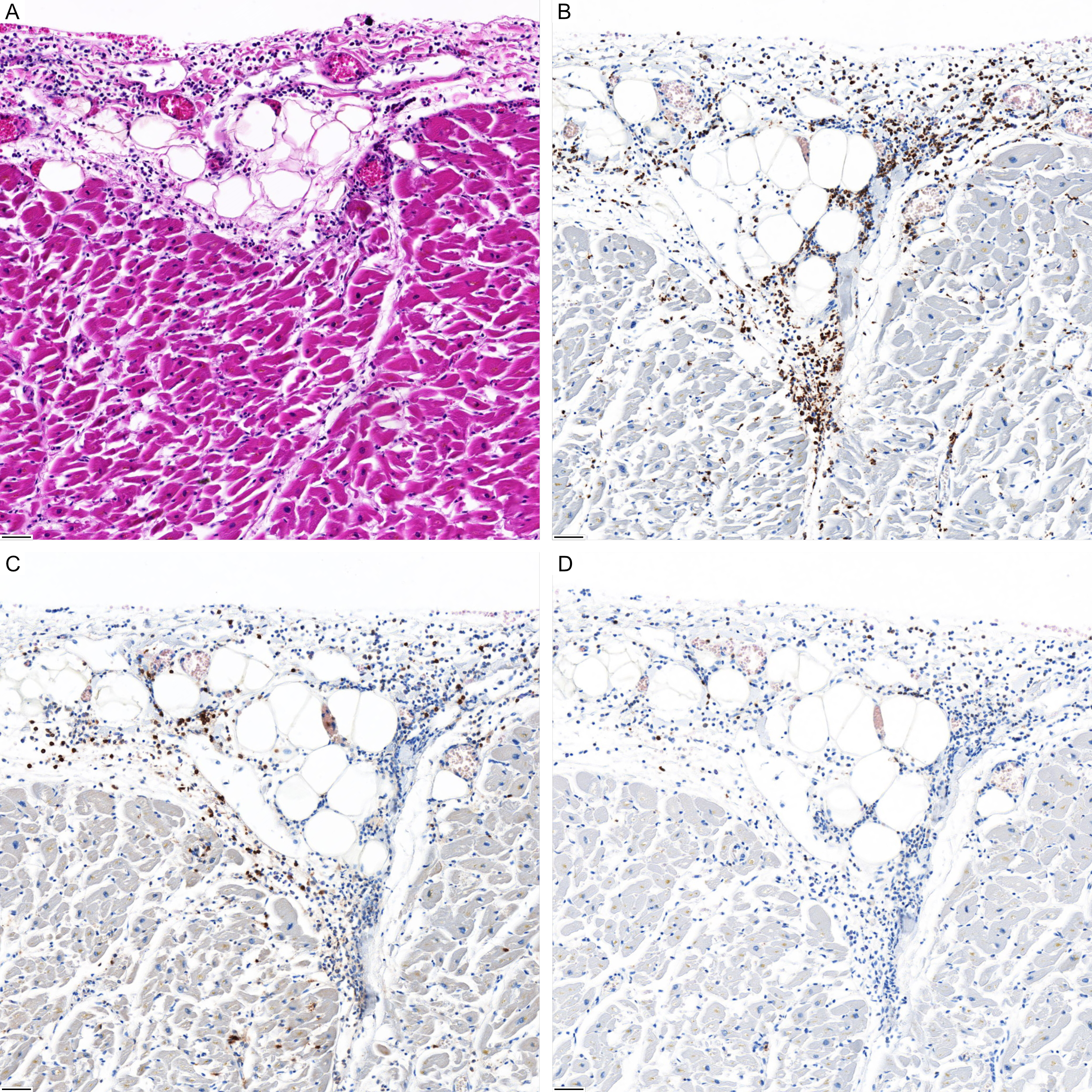

Supplement: Supplementary file 6 — Supplementary Figure 4: (A) Inflammatory infiltration of the epicardium of case 4, again showing (B) numerous CD68-positive macrophages and a T-cell infiltrate with (C) CD4-positive lymphocytes outnumbering (D) CD8-positive lymphocytes (TIFF 15031 KB) [file 392_2022_2129_MOESM6_ESM.tiff]
